# Effect of eflornithine on mutation frequency in temozolomide-treated U87MG cells

**DOI:** 10.18632/oncotarget.27782

**Published:** 2020-11-03

**Authors:** Noymi Yam, Jason Levin, Zhengzheng Bao, Wubin Qian, Victor A. Levin

**Affiliations:** ^1^Orbus Therapeutics, Inc., Palo Alto, CA, USA; ^2^Crown Bioscience, Inc., Santa Clara, CA, USA; ^3^Department of Neuro-Oncology, The University of Texas MD Anderson Cancer Center, Houston, TX, USA

**Keywords:** glioma, eflornithine, temozolomide, chemotherapy, secondary mutations

## Abstract

Treatment of infiltrative glioma presents a number of unique challenges due to poor penetration of typical chemotherapeutic agents into the infiltrating edge of tumors. The current chemotherapy options include nitrosoureas (e.g., lomustine) and the imidazotetrazine-class monofunctional DNA alkylating agent, temozolomide (TMZ). Both classes of drugs alkylate DNA and have relatively unrestricted passage from blood into brain where infiltrative tumor cells reside. Recent research indicates that secondary mutations detected in the RB and AKT-mTOR signaling pathways are linked to characteristics of recurrent tumors specific to TMZ-treated patients. It has been hypothesized that a decrease in rate of secondary mutations may result in delay of tumor recurrence. To that end, this study was designed to test viability of decreasing secondary mutations by disrupting the cell division cycle using eflornithine, a specific inhibitor of ornithine decarboxylase. U87MG glioblastoma cell line characterized by chromosomal abnormalities commonly attributed to primary cancers was used as a model for this study. The cells were subjected to TMZ treatment for 3 days followed by eflornithine (DFMO) treatment for 4 or 11 days. It was shown that TMZ significantly increased the frequency of mutations in U87MG glioblastoma cells while DFMO-treated cells showed mutation frequency statistically similar to that of the untreated cells on the respective treatment days. The findings of this study provide evidence to support the hypothesis that DFMO may inhibit progression of DNA mutations caused by alkylating chemotherapy agents, such as TMZ.

## INTRODUCTION

Malignant brain tumors in combination with other tumors of central nervous system (CNS) represent less than 2% of cancers in adults [1] and affect approximately 0.3% of world population [2]. Despite rare occurrence, brain cancers have a devastating impact on patients and society due to poor prognosis and limited treatment options. The five-year survival rate after diagnosis varies widely between approximately 5 and 80% with differences largely attributable to type and histology of tumors, patient age and genetic molecular markers.

The post-surgery treatment of malignant infiltrative gliomas, such as anaplastic astrocytoma and glioblastoma, usually involves a combination of radiotherapy and chemotherapy. Typical chemotherapy agents used are alkylating agents. Longest in use have been the nitrosoureas, such as carmustine (BCNU), lomustine (CCNU), and fotemustine, bifunctional alkylating agents that cross-link DNA, and more recently, the monofunctional alkylating agent temozolomide (TMZ). Another monofunctional alkylating agent is procarbazine that, while not approved by the FDA for brain tumor therapy has, none the less, been in use for the treatment of brain tumors for 40 years. Alkylating agents, and especially monofunctional alkylating agents, can produce unintended mutagenesis that in some cases results in secondary malignancies [3, 4].

Temozolomide (3,4-dihydro-3-methyl-4-oxoimidazo-[5,1-d]-1,2,3,4-tetrazin-8-carboximide, commonly abbreviated as TMZ), an imidazotetrazine-class chemotherapeutic agent, currently serves as a treatment of choice for both newly diagnosed and recurrent malignant brain tumors due to its high oral bioavailability and relatively efficient blood-brain barrier penetration [5, 6]. *In vivo*, TMZ acts as a prodrug, hydrolytically converting to its active metabolite monomethyl triazene 5-(3-methyltriazen-1-yl)-imidazole-4-carboxamide, which possesses high alkylating activity [7]. The active moiety is responsible for preferential methylation of guanine bases of the DNA and consecutive depletion of DNA repair protein O^6^-methylguanine DNA methyltransferase (MGMT) [8]. Mutagenic potential of TMZ has been observed in several studies [9, 10]. Recent research identified secondary mutations associated with use of TMZ that occur in anaplastic astrocytoma and glioblastoma [11, 12]. These studies extended earlier observations and studies of primary gliomas [13], unpaired recurrent tumors [14], and a cell culture model [15].

Eflornithine (D, L-2-(difluoromethyl) ornithine monohydrochloride monohydrate, often abbreviated as DFMO) is an enzyme-activated, irreversible inhibitor of the enzyme ornithine decarboxylase (ODC), the first and rate limiting enzyme in the biosynthesis of polyamines [16]. Inhibition of polyamine synthesis by eflornithine results in growth arrest of a number of malignant and nonmalignant mammalian cells and has been shown to inhibit the promotion and progression phases of carcinogenesis [17]. The goal of this study was to examine the effect of DFMO on the secondary mutations caused by alkylating agents, such as TMZ. Experimental model was based on U87MG glioblastoma cell line characterized by large number of chromosomal abnormalities commonly attributed to primary cancers [18]. Aggregate analysis was conducted to detect statistically significant trends in mutation frequency in cells subjected to different TMZ concentrations and subsequently treated with DFMO.

## RESULTS

**Figure 1:**
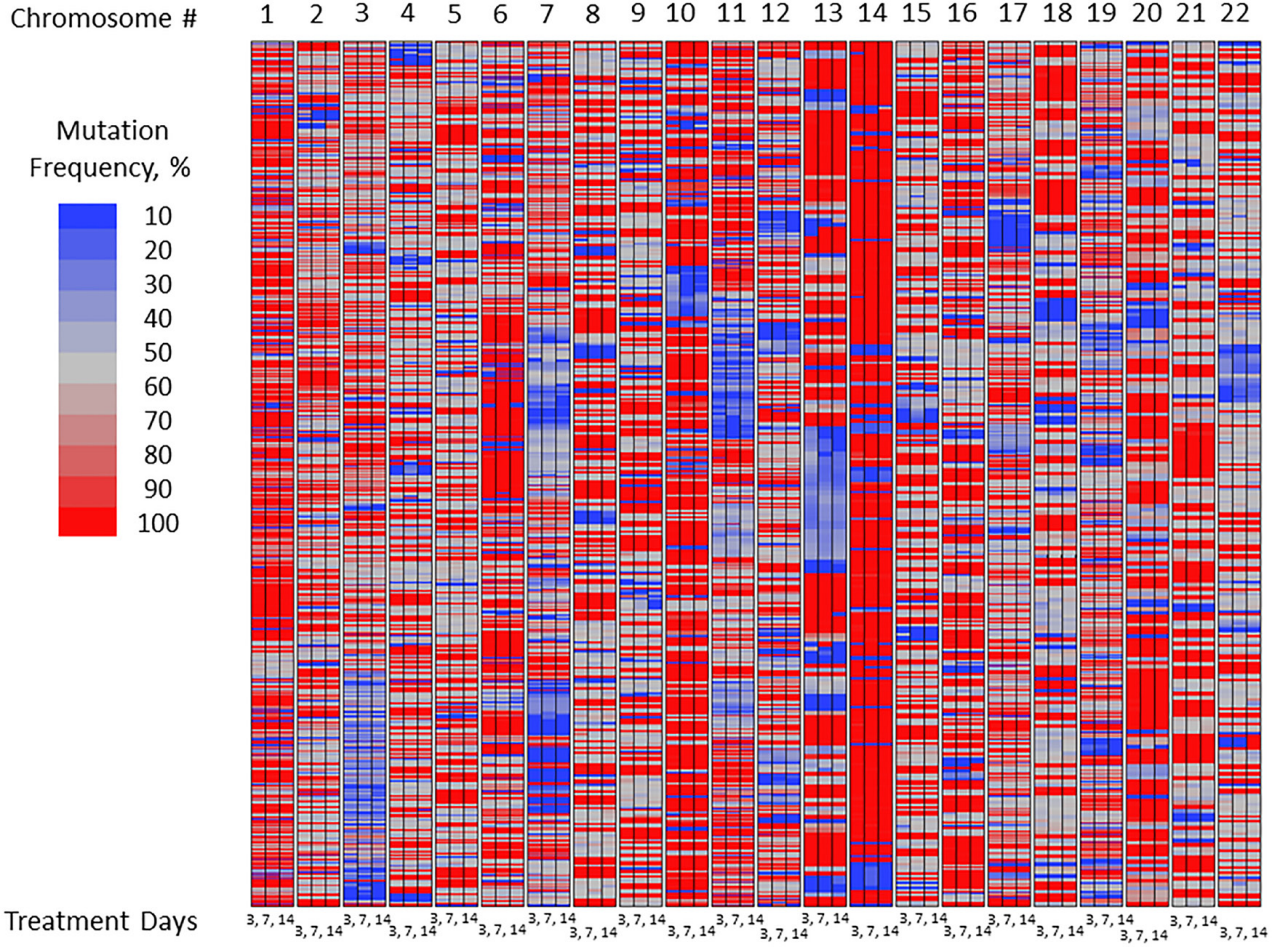
Mutation frequency in untreated U87MG glioma cells over the course of experiment: Exon-Seq analysis of the complete data set.

**Figure 2:**
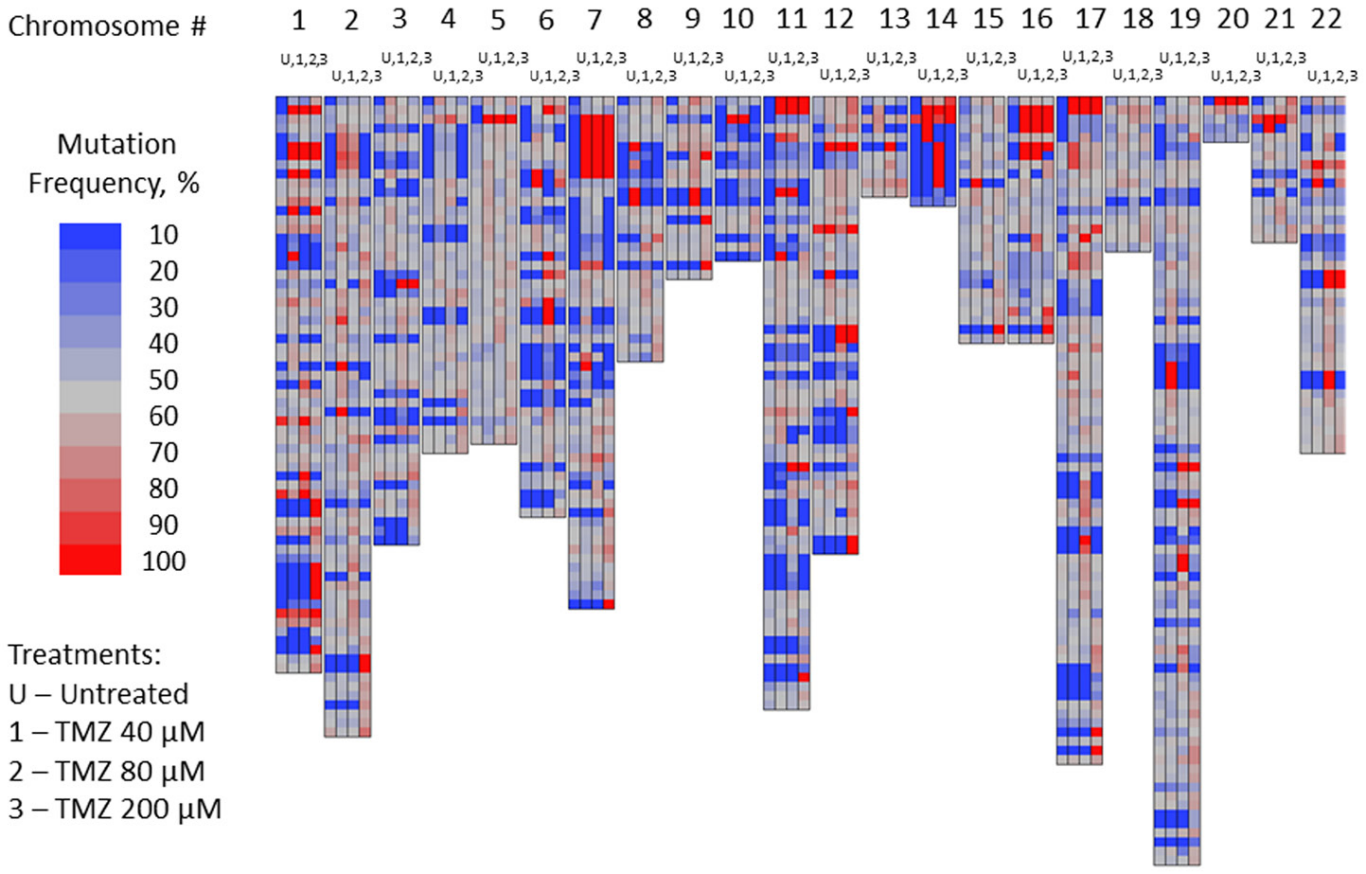
Mutation frequency in subset of nucleotides of U87MG glioma cells susceptible to TMZ-induced mutations: not-treated cells vs. cells treated with TMZ 40, 80 and 200 μM concentrations on day 3 of the treatment.

**Figure 3:**
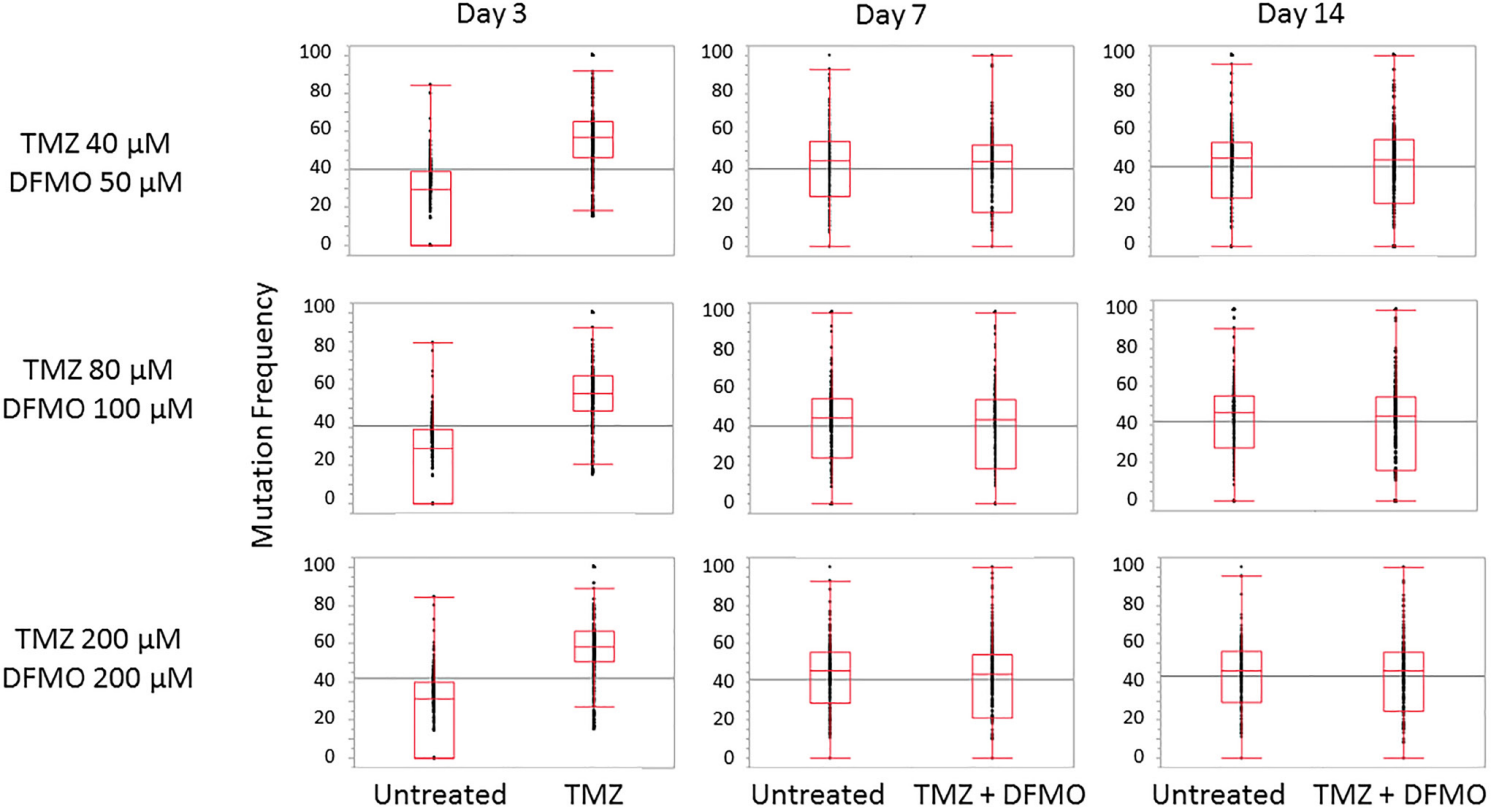
Trends in mutation frequency in nucleotides susceptible to TMZ -induced mutations. Mean mutation frequency increase observed after 3 days of treatment with TMZ at three different concentrations (40, 80 and 200 μM) is in the range of 34–37%. Subsequent treatment with 50, 100 and 200 μM DFMO yields mutation frequency statistically equivalent to that of untreated cells.

**Figure 4:**
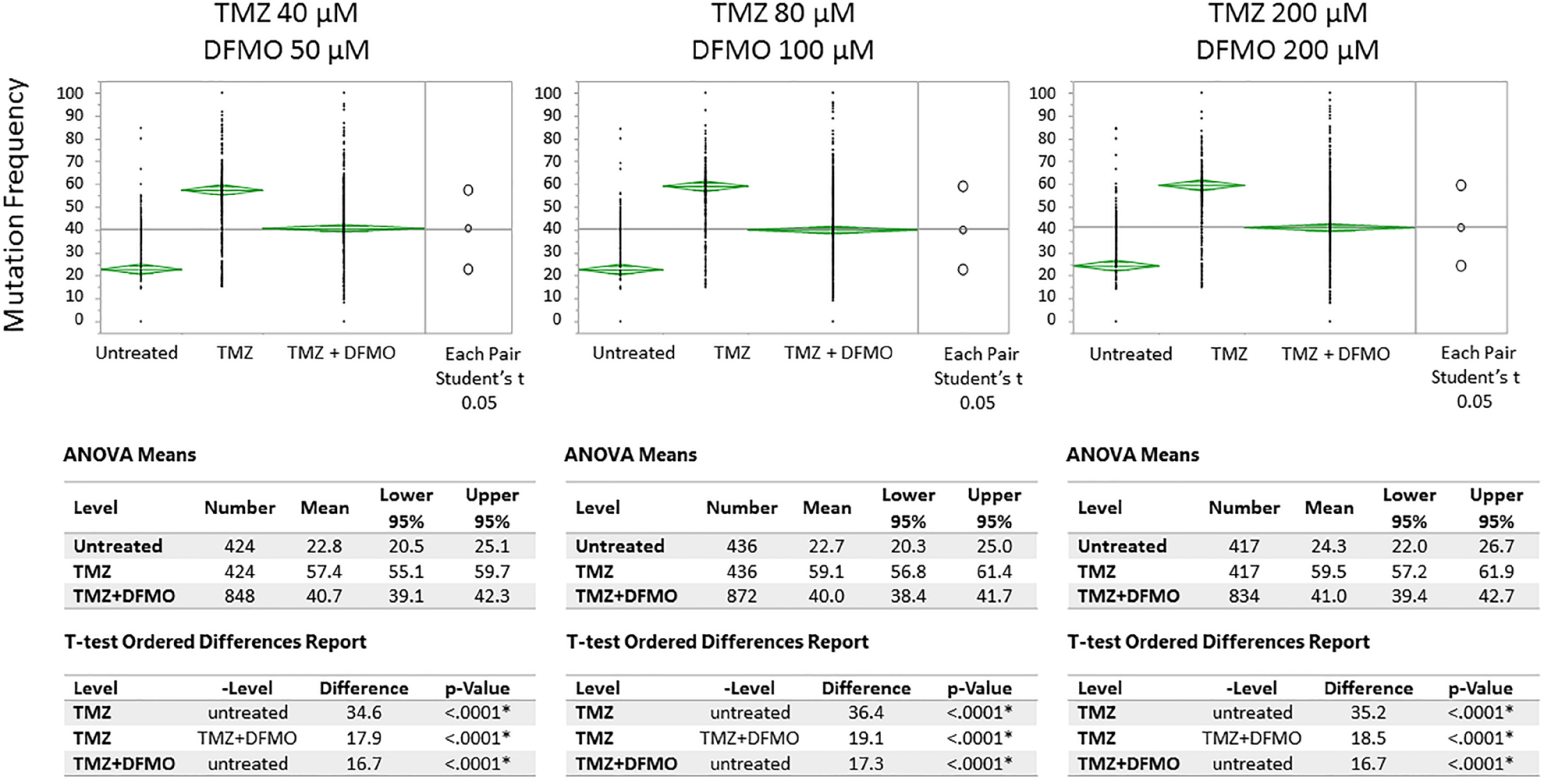
Means/ANOVA analysis of mutation frequency in U87MG glioma cell nucleotides susceptible to TMZ-induced mutations. Statistically significant increase of mutation frequency demonstrated for TMZ-treated vs. untreated cells on Day 3. Subsequent treatment with DFMO at each level of TMZ showed significantly lower level of mutation frequency on days 7 and 14 (day 7 and 14 data shown in aggregate)^1^. ^1^Note: the top and bottom of each diamond represent the confidence interval for each group. The mean line across the middle of each diamond represents the group mean. The multiple comparison test is illustrated by a comparison circles plot on the right of the graph and the degree of intersection of the circles shows whether the group means are significantly different. Non-intersecting circles show statistically significant differences.

Exon-Seq analysis confirmed inherently high levels of mutations in the untreated U87MG glioblastoma cells over the course of experiment (Figure 1). As part of preliminary data analysis, it was necessary to identify the subset of nucleotides characterized by a relatively low initial mutation frequency, on which the mutagenic effect of TMZ could be examined. Further data analysis was performed to detect the nucleotides susceptible to TMZ-induced mutations using a 15% threshold shift in mutation rate as a basic criterion (Figure 2). The sub-groups of nucleotides were defined for each TMZ concentration as well as for a combination of concentrations at different time points. The mutation frequencies of the resulting subsets were analyzed using an analysis of variance (ANOVA) and Student’s *t*-test. The comparative analysis showed that exposure to TMZ caused statistically significant increase in cell mutations across the range of concentrations. The mean mutation frequency on day 3 of exposure increased from 23% for the untreated cells to 57%, 59% and 60% for the TMZ concentrations of 40, 80 and 200 μM, respectively (Table 1). Interestingly, when the TMZ-exposed cells from the respective subsets were further treated with 50, 100 and 200 μM DFMO solutions, the nucleotides showed statistically lower mutation frequencies with means of 39–42% on days 7 and 14 of exposure. These lower mutation frequencies of DFMO-treated cells were statistically equivalent to that of the untreated cells on the respective treatment days (Figure 3). The same trends were also confirmed by aggregate analysis of the combined time point data for each type of treatment (Figure 4). For each pair of TMZ and DFMO concentrations, the statistically significant mutation frequency increase was observed on day 3 of TMZ treatment while the rate of mutation frequency was markedly decreased on days 7 and 14 for the DFMO-treated nucleotide subsets.

**Table 1:** Mean mutation frequency in glioblastoma cell line model

TMZ Treatment Days 1–3	Treatment Days 4–14	Mean Mutation Frequency		
		T = 3 days	T = 7 days	T = 14 days
Untreated control	No treatment	23%	42%	43%
0	DFMO 50 μM	23%	39%	38%
0	DFMO 100 μM	23%	40%	39%
0	DFMO 200 μM	23%	41%	42%
TMZ 40 μM	DFMO 50 μM	57%	40%	42%
TMZ 80 μM	DFMO 100 μM	59%	39%	41%
TMZ 200 μM	DFMO 200 μM	60%	40%	42%

**Figure 5:**
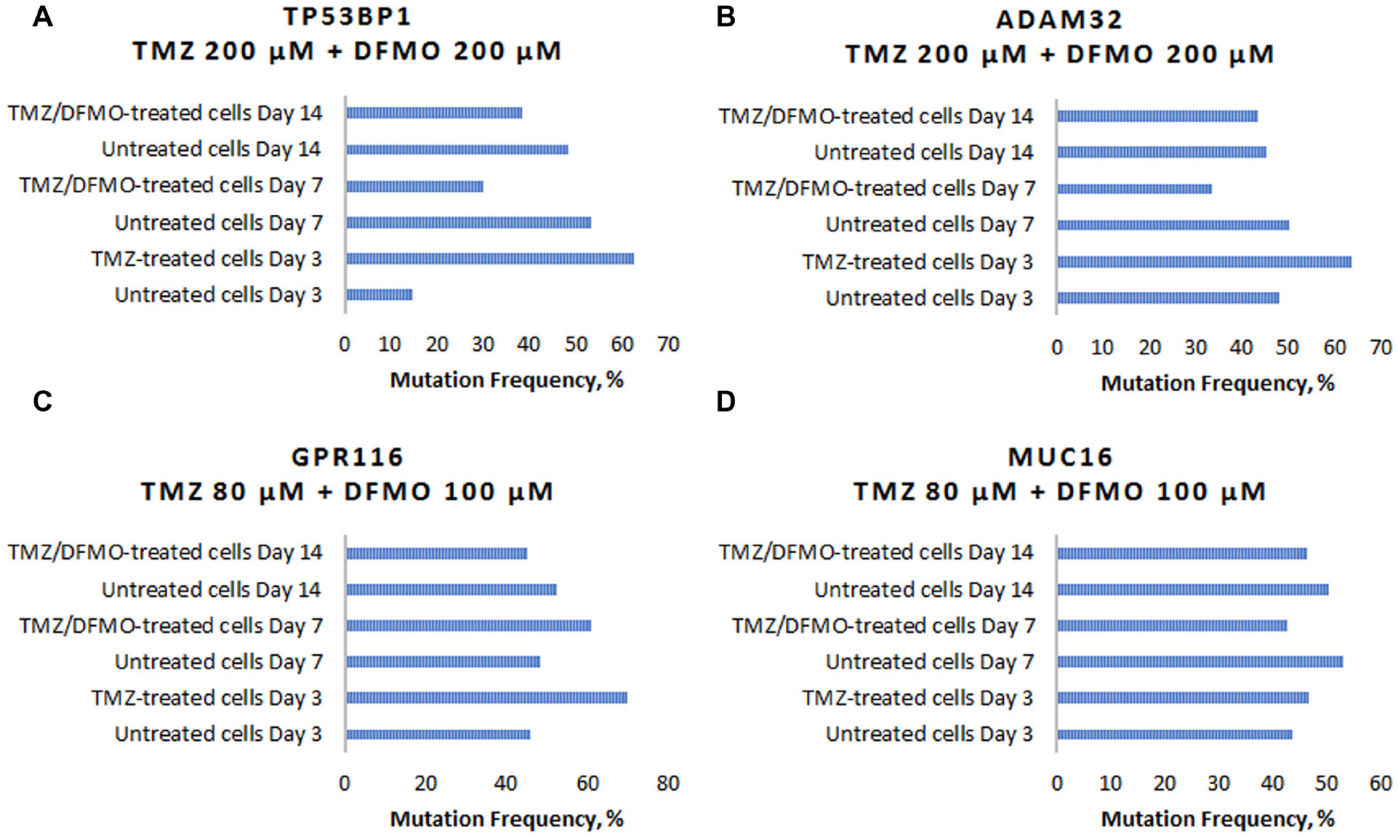
Examples of TMZ-induced mutation frequency increase and subsequent mutation-inhibiting action of DFMO for genes involved in carcinogenesis. (**A**) Changes in C/T mutation frequency in TP53BP1 gene. (**B**) Changes in G/A mutation frequency in ADAM32 gene. (**C**) Changes in C/T mutation in GPR116 gene. (**D**) Changes in T/A mutation in MUC16 gene.

To illustrate the effect of TMZ-induced mutagenesis and subsequent mutation-inhibiting action of DFMO, four cases are reviewed for the specific genes known to play an important role in carcinogenesis: TP53BP1, ADAM32, GPR116 and MUC16. TP53BP1, also known as Tumor Suppressor P53-Binding Protein 1, is located on chromosome 15 which encodes a protein that functions in the DNA double-strand break repair pathway choice (apoptosis pathway), promoting non-homologous end joining pathways, and limiting homologous recombination. This protein plays multiple roles in the DNA damage response, including promoting checkpoint signaling following DNA damage, acting as a scaffold for recruitment of DNA damage response proteins to damaged chromatin, and promoting NHEJ pathways by limiting end resection following a double-strand break. TP53 has been linked to mutations present in post-TMZ treated gliomas. In the experiment, the U87MG cells showed C/T mutation in TP53BP1 gene location chr15: 43,762,196. The frequency of this mutation in the untreated cells on day 3 was 15%. Comparatively, the cells subjected to TMZ treatment at 200 μM concentration showed the mutation frequency of 62% on day 3. As the experiment continued, the untreated cells reached mutation frequency of 53% on day 7 and 48% on day 14. The cells which were treated with TMZ for 3 days and then subjected to DFMO treatment at 200 μM concentration showed the reduced mutation rates of 30% and 38% at 7 and 14 days, respectively (Figure 5A).

The second example, ADAM32, also known as a disintegrin and metalloproteinase domain 32 is located on chromosome 8. ADAM 32 is a member of the disintegrin family of membrane anchored proteins, which play a role in diverse biological processes such as brain development, fertilization, tumor development and inflammation. It is present in reproductive organs. Mutations in this gene have been associated with hypermutation in gliomas. In this experiment, the U87MG cells showed G/A mutation in ADAM 32 gene location chr8: 38,964,647. The frequency of this mutation in the untreated cells on day 3 was 48%, while the mutation frequency of the cells subjected to TMZ treatment at 200 μM concentration reached 64% on day 3. As the experiment continued, the untreated cells mutation remained at similar rate of 50% on day 7 and 45% on day 14. The DFMO treatment after TMZ exposure resulted in the reduced mutation rates of 33% and 43% on days 7 and 14, respectively (Figure 5B).

GPR116, also known as ADGRF5, is located on chromosome 6 and is a G protein-coupled receptor 116. GPRs are cell surface receptors that activate guanine-nucleotide binding proteins upon the binding of a ligand. GPRs may play a role in neuron survival through activation of a downstream signaling pathway involving the PI3, Akt and MAP kinases. GPR112 has been identified as a hypermutating gene in gliomas after TMZ treatment. GPRs are involved in cellular proliferation and evading apoptosis. The U87MG cells showed C/T mutation in GPR116 gene location chr6: 46,867,771. The frequency of this mutation in the untreated cells on day 3 was 46%. The cells subjected to TMZ treatment at 80 μM concentration showed the mutation frequency of 70% on day 3. As the experiment continued, the untreated cells mutation reached 48% on day 7 and 52% on day 14. The cells which were treated with TMZ for 3 days and then subjected to DFMO treatment at 100 μM concentration showed the mutation rates of 61% and 45% at 7 and 14 days, respectively (Figure 5C).

MUC16 located on chromosome 19, encodes a protein that is a member of the mucin family. This protein is thought to play a role in forming a barrier, protecting epithelial cells from pathogens. Products of this gene have been used as a marker for different cancers (e.g., ovarian carcinoma), with higher expression levels associated with poorer outcomes. MUC16 is involved in cell migration and is implicated in mutations present in post-TMZ treated gliomas. The T/A mutation in GPR116 gene location chr19: 9,071,763 showed the frequency of 43% for the untreated cells on day 3. The cells subjected to TMZ treatment at 80 μM concentration showed the mutation frequency of 64% on day 3. As the experiment continued, the untreated cells mutation rate reached 58% on day 7 and 61% on day 14. The cells which were treated with TMZ for 3 days and then subjected to DFMO treatment at 100 μM concentration showed the mutation rates of 49% and 47% at 7 and 14 days, respectively (Figure 5D).

## DISCUSSION

In our studies, exposure to TMZ caused a significant increase in frequency of cancer-related mutations in U87MG glioblastoma cells as measured by quantifying known nucleotide polymorphisms using Exon-Seq analysis. Subsequent treatment with DFMO caused a statistically significant decrease in the mutation frequency compared to TMZ-treated cells. The effects were shown for three different TMZ and DFMO concentrations as well as for the combined data set. While the TMZ-induced mutagenesis has been extensively explored in recent research, the inhibitory action of DFMO leading to decrease in mutation frequency has not been reported before.

It was previously shown that hypermutations induced by the alkylating agents can increase resistance to a number of anti-neoplastic drugs and worsen prognosis in a significant fraction of patients treated with TMZ [15, 19, 20]. The existence of TMZ-induced mutagenesis in glioma and its association with progression of the disease from the early stages to the most serious form, glioblastoma multiforme, has been recognized [21, 22]. Mutations known to be linked to treatment with TMZ were shown to occur in the retinoblastoma protein (RB) and AKT-motor pathways and were correlated with tumor progression. In further support of the importance of mutation to transformation of low- and mid-grade gliomas to more malignant tumor grades, is a study published in 2013 [11]. The hypothesis of this study was that therapies for recurrent/progressive gliomas failed because the genomic alterations driving the growth of recurrences were distinct from those in the initial tumor. In the study, the exomes of 23 initial low-grade gliomas were sequenced and recurrent tumors resected from the same patients. It was found that the three genes most mutated in WHO grade 2 gliomas at initial diagnosis were: IDH1 in 100% (23/23), TP53 in 83% (19/23), and ATRX in 78% (18/23) in the cohort studied. Interestingly, in 43% of cases, at least half of the mutations in the initial tumor were undetected at tumor recurrence suggesting that recurrent tumors may be seeded by cells derived from the initial tumor at a very early stage of their evolution. Of additional interest was the observation that tumors from 60% of patients treated with adjuvant TMZ chemotherapy followed an alternative evolutionary path to high-grade glioma: these tumors showed hypermutation and harbored driver mutations in the RB and AKT-mTOR pathways that bore the signature of TMZ-induced mutagenesis.

Given that the gliomas generally follow a path of mutation if they recur or progress, one approach to control progression would be to mitigate the rate and extent of mutations these tumors can express. In the current study, we explored the concept of disrupting the TMZ-induced hypermutations by interrupting the cell division cycle using DFMO. The existing pharmaceutical applications of DFMO for treatment of African trypanosomiasis and hirsutism rely on its impact on cell division cycle by inhibition of ODC [23], an irreversible metabolic process with well-established mechanism of action [24]. The reaction catalyzed by ornithine decarboxylase is the first and committed step in the synthesis of polyamines, especially putrescine, spermine, and spermidine. Polyamines have been found in high levels in many tumor cells and appear to support cell growth that is essential for the multistep process of cancer development [25, 26]. Lack of ornithine decarboxylase activity has been demonstrated to induce apoptosis and cell death [27, 28]. Studies over many years have shown that the activity of ODC increases with grade of malignancy for adenocarcinomas of the breast, lung, and colon [29–37] as well as for gliomas and medulloblastoma tumors [38–43]. Of considerable interest is the observation that there is a relationship between DFMO activity and ODC levels since patients with relatively low levels of ODC appear to respond better to DFMO and DFMO-nitrosourea combinations [44–47]. It is well known that ODC activity is directly correlated with the grade of the glioma, with higher grades of glioma having higher ODC levels [44]. Published clinical studies affirmed evidence for higher clinical activity of DFMO in anaplastic astrocytoma than in glioblastoma [46, 47]. While the findings of this study provide evidence to support the hypothesis that DFMO may inhibit progression of DNA mutations caused by alkylating chemotherapy agents, they may also suggest that one of the benefits of DFMO in the prior clinical trials have been inhibition of *de novo* mutations by inducing G1-arrest in glioma and subsequently increasing intracellular p21 and p27kip-1 proteins [48, 49] thus impacting tumor cell mutation rates and inhibiting progression to more malignant tumor phenotypes.

## MATERIALS AND METHODS

U87MG glioblastoma cells were procured from American Type Culture Collection (ATCC). Frozen cells were rapidly thawed in a 37°C water bath, then slowly diluted using pre-warmed growth medium and plated at high density to optimize recovery. The cells were then harvested and placed in T75 flasks with final cell number of 4 × 10^6^ cells/flask. The flasks were incubated overnight in humidified incubator at 37°C with 5% CO_2_.

A preliminary experiment was performed to determine the TMZ concentrations sufficient to provide a viable range of exposure while maximizing cell survival to enable quantitation of mutation frequency by Exon-Seq analysis. The resulting TMZ concentrations for three levels of this experiment were 40, 80 and 200 μM, respectively.

The test solutions were applied to the incubated U87MG cells at 37.5 μL per flask. Control set of flasks was treated with DMSO at concentration of 0.25% v/v in culture medium and the samples were tested at 3, 7 and 14 days to provide baseline mutation frequency for the U87MG cell line. In parallel, another set of flasks with U87MG glioblastoma cells, pre-conditioned identically to the control was separated into three subsets, each treated with different concentration of TMZ: 40, 80 or 200 μM. The cells were incubated with TMZ solutions for 3 days and then samples from each subset were tested to assess the effect of TMZ as single agent on cell mutation frequency. Following the TMZ treatment at three different concentrations for 3 days, the cells were subjected to the DFMO treatments at 50, 100 and 200 μM concentrations, respectively, for additional 11 days providing total of 14 days of total treatment exposure. Lastly, the untreated cells were used to create three additional subsets that were not treated for the initial 3 days of experiment and were subjected only to DFMO treatments at 50, 100 or 200 μM concentrations.

After exposure, the cells were placed into new T75 flasks to obtain cell density of 2 × 10^6^ cells/flask and returned to the incubator for an additional 7 days. After additional incubation, the cells were re-suspended in PBS and centrifuged for 5 minutes at approximately 1000 rpm. The supernatant was removed, and the cell pellet stored at –80°C before it was used for Exon-Seq analysis. The cells harvested at different time points were counted using a Count-Star automated cell counter.

Exon-Seq analysis was performed per optimized protocol for Illumina paired-end multiplexed library preparation using the SureSelect^XT^ Library Prep and Capture System (Agilent Technologies). DNA was extracted from cells (0.5 × 10^6^) using commercial DNA kits according to the manufacturer`s instructions. For the purposes of library preparation, 300 ng genomic DNA concentrations were measured with the Qubit 2.0 fluorometer dsDNA HS Assay (Thermo Fisher Scientific) and sheared with the Covaris LE220 Sonicator (Covaris) to target 150–200 bp average size. DNA libraries were prepared using the Sureselect^XT^ reagent kit (Agilent Technologies). The 3′ and 5′ overhangs on the DNA fragments were repaired using End repair mix (a component of the Sureselect^XT^ kit) and purified using Agencourt AMPure ^XP^ Beads (Beckman). The purified fragments were added with “A” tail using A tailing Mix and then ligated with an adapter using DNA ligase. The adapter-ligated DNA fragments were amplified with Herculase II Fusion DNA Polymerase (Agilent). Finally, the pre-capture libraries containing exome sequences were captured using the SureSelect capture library kit (Agilent). For Illumina sequencing, DNA concentration of the enriched sequencing libraries was measured with the Qubit 2.0 fluorometer dsDNA HS Assay (Thermo Fisher Scientific). Size distribution of the resulting sequencing libraries was analyzed using the Agilent BioAnalyzer 2100 (Agilent). The libraries were used in cluster formation on an Illumina cBOT cluster generation system with HiSeq PE Cluster Kits (Illumina). Paired-end sequencing was performed using an Illumina HiSeq system following Illumina-provided protocols for 2 × 150 paired-end sequencing. This PCR based amplification method provided a relative number (e.g., fluorescence intensity) for each nucleotide polymorphism detected. This represents the comparative frequency of each mutation across samples (e.g., mutation frequency). The mutation frequency results were analyzed for 13,040 nucleotide polymorphisms across 6,455 genes that have been previously identified as potentially cancer related based on their description in the Catalogue of Somatic Mutations in Cancer (COSMIC) database.

## Abbreviations

DFMO: eflornithine
TMZ: temozolomide
CCNU: lomustine
BCNU: carmustine
CNS: central nervous system
MGMT: O^6^-methylguanine DNA methyltransferase
ATCC: American Type Culture Collection
COSMIC: Catalogue of Somatic Mutations in Cancer

## References

[1] Siegel RL , Miller KD , Jemal A . Cancer statistics, 2019. CA Cancer J Clin. 2019; 69:7–34. 10.3322/caac.21551. 30620402

[2] Central Brain Tumor Registry of the United States. CBTRUS 2018 Fact Sheet. 2018.

[3] Sanderson BJ , Shield AJ . Mutagenic damage to mammalian cells by therapeutic alkylating agents. Mutat Res. 1996; 355:41–57. 10.1016/0027-5107(96)00021-8. 8781576

[4] Pletsa V , Valavanis C , van Delft JH , Steenwinkel MJ , Kyrtopoulos SA . DNA damage and mutagenesis induced by procarbazine in lambda lacZ transgenic mice: evidence that bone marrow mutations do not arise primarily through miscoding by O6-methylguanine. Carcinogenesis. 1997; 18:2191–2196. 10.1093/carcin/18.11.2191. 9395220

[5] Zhang J , Stevens MF , Bradshaw TD . Temozolomide: mechanisms of action, repair and resistance. Curr Mol Pharmacol. 2012; 5:102–114. 10.2174/1874467211205010102. 22122467

[6] Schreck KC , Grossman SA . Role of Temozolomide in the Treatment of Cancers Involving the Central Nervous System. Oncology (Williston Park). 2018; 32:555–60,69. 30474103

[7] Reid JM , Stevens DC , Rubin J , Ames MM . Pharmacokinetics of 3-methyl-(triazen-1-yl)imidazole-4-carboximide following administration of temozolomide to patients with advanced cancer. Clin Cancer Res. 1997; 3:2393–2398. 9815639

[8] Stupp R , Mason WP , van den Bent MJ , Weller M , Fisher B , Taphoorn MJ , Belanger K , Brandes AA , Marosi C , Bogdahn U , Curschmann J , Janzer RC , Ludwin SK , et al; European Organisation for Research and Treatment of Cancer Brain Tumor and Radiotherapy Groups; National Cancer Institute of Canada Clinical Trials Group. Radiotherapy plus concomitant and adjuvant temozolomide for glioblastoma. N Engl J Med. 2005; 352:987–996. 10.1056/NEJMoa043330. 15758009

[9] Geiger H , Schleimer D , Nattamai KJ , Dannenmann SR , Davies SM , Weiss BD . Mutagenic potential of temozolomide in bone marrow cells *in vivo* . Blood. 2006; 107:3010–3011. 10.1182/blood-2005-09-3649. 16554488

[10] van Thuijl HF , Mazor T , Johnson BE , Fouse SD , Aihara K , Hong C , Malmström A , Hallbeck M , Heimans JJ , Kloezeman JJ , Stenmark-Askmalm M , Lamfers ML , Saito N , et al. Evolution of DNA repair defects during malignant progression of low-grade gliomas after temozolomide treatment. Acta Neuropathol. 2015; 129:597–607. 10.1007/s00401-015-1403-6. 25724300 PMC4482618

[11] Johnson BE , Mazor T , Hong C , Barnes M , Aihara K , McLean CY , Fouse SD , Yamamoto S , Ueda H , Tatsuno K , Asthana S , Jalbert LE , Nelson SJ , et al. Mutational analysis reveals the origin and therapy-driven evolution of recurrent glioma. Science. 2014; 343:189–193. 10.1126/science.1239947. 24336570 PMC3998672

[12] Daniel P , Sabri S , Chaddad A , Meehan B , Jean-Claude B , Rak J , Abdulkarim BS . Temozolomide Induced Hypermutation in Glioma: Evolutionary Mechanisms and Therapeutic Opportunities. Front Oncol. 2019; 9:41. 10.3389/fonc.2019.00041. 30778375 PMC6369148

[13] Hunter C , Smith R , Cahill DP , Stephens P , Stevens C , Teague J , Greenman C , Edkins S , Bignell G , Davies H , O’Meara S , Parker A , Avis T , et al. A hypermutation phenotype and somatic MSH6 mutations in recurrent human malignant gliomas after alkylator chemotherapy. Cancer Res. 2006; 66:3987–3991. 10.1158/0008-5472.CAN-06-0127. 16618716 PMC7212022

[14] Cancer Genome Atlas Research Network. Comprehensive genomic characterization defines human glioblastoma genes and core pathways. Nature. 2008; 455:1061–1068. 10.1038/nature07385. 18772890 PMC2671642

[15] Bodell WJ , Gaikwad NW , Miller D , Berger MS . Formation of DNA adducts and induction of lacI mutations in Big Blue Rat-2 cells treated with temozolomide: implications for the treatment of low-grade adult and pediatric brain tumors. Cancer Epidemiol Biomarkers Prev. 2003; 12:545–551. 12815001

[16] Franklin TJ , Snow GA . Biochemistry and molecular biology of antimicrobial drug action. New York: Springer; 2005 10.1007/0-387-27566-5.

[17] Abbruzzese JL . Gastrointestinal oncology. Oxford, New York: Oxford University Press; 2004.

[18] Clark MJ , Homer N , O’Connor BD , Chen Z , Eskin A , Lee H , Merriman B , Nelson SF . U87MG decoded: the genomic sequence of a cytogenetically aberrant human cancer cell line. PLoS Genet. 2010; 6:e1000832. 10.1371/journal.pgen.1000832. 20126413 PMC2813426

[19] Yip S , Miao J , Cahill DP , Iafrate AJ , Aldape K , Nutt CL , Louis DN . MSH6 mutations arise in glioblastomas during temozolomide therapy and mediate temozolomide resistance. Clin Cancer Res. 2009; 15:4622–4629. 10.1158/1078-0432.CCR-08-3012. 19584161 PMC2737355

[20] Greenman C , Stephens P , Smith R , Dalgliesh GL , Hunter C , Bignell G , Davies H , Teague J , Butler A , Stevens C , Edkins S , O’Meara S , Vastrik I , et al. Patterns of somatic mutation in human cancer genomes. Nature. 2007; 446:153–158. 10.1038/nature05610. 17344846 PMC2712719

[21] Mazor T , Pankov A , Johnson BE , Hong C , Hamilton EG , Bell RJA , Smirnov IV , Reis GF , Phillips JJ , Barnes MJ , Idbaih A , Alentorn A , Kloezeman JJ , et al. DNA Methylation and Somatic Mutations Converge on the Cell Cycle and Define Similar Evolutionary Histories in Brain Tumors. Cancer Cell. 2015; 28:307–317. 10.1016/j.ccell.2015.07.012. 26373278 PMC4573399

[22] Wang Q , Hu B , Hu X , Kim H , Squatrito M , Scarpace L , deCarvalho AC , Lyu S , Li P , Li Y , Barthel F , Cho HJ , Lin YH , et al. Tumor Evolution of Glioma-Intrinsic Gene Expression Subtypes Associates with Immunological Changes in the Microenvironment. Cancer Cell. 2018; 33:152. 10.1016/j.ccell.2017.12.012. 29316430 PMC5892424

[23] LoGiudice N , Le L , Abuan I , Leizorek Y , Roberts SC . Alpha-Difluoromethylornithine, an Irreversible Inhibitor of Polyamine Biosynthesis, as a Therapeutic Strategy against Hyperproliferative and Infectious Diseases. Med Sci (Basel). 2018; 6:12. 10.3390/medsci6010012. 29419804 PMC5872169

[24] Poulin R , Lu L , Ackermann B , Bey P , Pegg AE . Mechanism of the irreversible inactivation of mouse ornithine decarboxylase by alpha-difluoromethylornithine. Characterization of sequences at the inhibitor and coenzyme binding sites. J Biol Chem. 1992; 267:150–158. 1730582

[25] Gerner EW , Meyskens FL Jr . Polyamines and cancer: old molecules, new understanding. Nat Rev Cancer. 2004; 4:781–792. 10.1038/nrc1454. 15510159

[26] Casero RA Jr , Murray Stewart T , Pegg AE . Polyamine metabolism and cancer: treatments, challenges and opportunities. Nat Rev Cancer. 2018; 18:681–695. 10.1038/s41568-018-0050-3. 30181570 PMC6487480

[27] Askew DS , Ashmun RA , Simmons BC , Cleveland JL . Constitutive c-myc expression in an IL-3-dependent myeloid cell line suppresses cell cycle arrest and accelerates apoptosis. Oncogene. 1991; 6:1915–1922. 1923514

[28] Huang Y , Hager ER , Phillips DL , Dunn VR , Hacker A , Frydman B , Kink JA , Valasinas AL , Reddy VK , Marton LJ , Casero RA Jr , Davidson NE . A novel polyamine analog inhibits growth and induces apoptosis in human breast cancer cells. Clin Cancer Res. 2003; 9:2769–2777. 12855657 PMC3625930

[29] Glikman P , Vegh I , Pollina MA , Mosto AH , Levy CM . Ornithine decarboxylase activity, prolactin blood levels, and estradiol and progesterone receptors in human breast cancer. Cancer. 1987; 60:2237–2243. 10.1002/1097-0142(19871101)60:9<2237::AID-CNCR2820600923>3.0.CO;2-J. 3440234

[30] Thomas T , Kiang DT , Janne OA , Thomas TJ . Variations in amplification and expression of the ornithine decarboxylase gene in human breast cancer cells. Breast Cancer Res Treat. 1991; 19:257–267. 10.1007/BF01961162. 1663805

[31] Manni A , Wechter R , Wei L , Heitjan D , Demers L . Phenotypic features of breast cancer cells overexpressing ornithine- decarboxylase. J Cell Physiol. 1995; 163:129–136. 10.1002/jcp.1041630115. 7896889

[32] Manni A , Mauger D , Gimotty P , Badger B . Prognostic influence on survival of increased ornithine decarboxylase activity in human breast cancer. Clin Cancer Res. 1996; 2:1901–1906. 9816147

[33] Mimori K , Mori M , Shiraishi T , Tanaka S , Haraguchi M , Ueo H , Shirasaka C , Akiyoshi T . Expression of ornithine decarboxylase mRNA and c-myc mRNA in breast tumours. Int J Oncol. 1998; 12:597–601. 10.3892/ijo.12.3.597. 9472098

[34] Cañizares F , Salinas J , de las Heras M , Diaz J , Tovar I , Martinez P , Peñafiel R . Prognostic value of ornithine decarboxylase and polyamines in human breast cancer: correlation with clinicopathologic parameters. Clin Cancer Res. 1999; 5:2035–2041. 10473083

[35] Mohan RR , Challa A , Gupta S , Bostwick DG , Ahmad N , Agarwal R , Marengo SR , Amini SB , Paras F , MacLennan GT , Resnick MI , Mukhtar H . Overexpression of ornithine decarboxylase in prostate cancer and prostatic fluid in humans. Clin Cancer Res. 1999; 5:143–147. 9918212

[36] LaMuraglia GM , Lacaine F , Malt RA . High ornithine decarboxylase activity and polyamine levels in human colorectal neoplasia. Ann Surg. 1986; 204:89–93. 10.1097/00000658-198607000-00013. 3729588 PMC1251228

[37] Berdinskikh NK , Ignatenko NA , Zaletok SP , Ganina KP , Chorniy VA . Ornithine decarboxylase activity and polyamine content in adenocarcinomas of human stomach and large intestine. Int J Cancer. 1991; 47:496–498. 10.1002/ijc.2910470404. 1995479

[38] Scalabrino G , Modena D , Ferioli ME , Puerari M , Luccarelli G . Degrees of malignancy in human primary central nervous system tumors: ornithine decarboxylase levels as better indicators than adenosylmethionine decarboxylase levels. J Natl Cancer Inst. 1982; 68:751–754. 6951085

[39] Scalabrino G , Ferioli ME . Degree of enhancement of polyamine biosynthetic decarboxylase activities in human tumors: a useful new index of degree of malignancy. Cancer Detect Prev. 1985; 8:11–16. 4064029

[40] Ernestus RI , Röhn G , Schröder R , Klug N , Hossmann KA , Paschen W . Activity of ornithine decarboxylase (ODC) and polyamine levels as biochemical markers of malignancy in human brain tumors. Acta Histochem Suppl. 1992; 42:159–164. 1584960

[41] Ernestus RI , Röhn G , Schröder R , Els T , Lee JY , Klug N , Paschen W . Polyamine metabolism in gliomas. J Neurooncol. 1996; 29:167–174. 10.1007/BF00182140. 8858522

[42] Ernestus RI , Röhn G , Schröder R , Els T , Klekner A , Paschen W , Klug N . Polyamine metabolism in brain tumours: diagnostic relevance of quantitative biochemistry. J Neurol Neurosurg Psychiatry. 2001; 71:88–92. 10.1136/jnnp.71.1.88. 11413269 PMC1737459

[43] Levin VA , Jochec JL , Shantz LM , Koch PE , Pegg AE . Tissue-based assay for ornithine decarboxylase to identify patients likely to respond to difluoromethylornithine. J Histochem Cytochem. 2004; 52:1467–1474. 10.1369/jhc.4A6358.2004. 15505341 PMC3957822

[44] Levin VA , Jochec JL , Shantz LM , Aldape KD . Relationship between ornithine decarboxylase levels in anaplastic gliomas and progression-free survival in patients treated with DFMO-PCV chemotherapy. Int J Cancer. 2007; 121:2279–2283. 10.1002/ijc.22914. 17582600

[45] Levin VA , Uhm JH , Jaeckle KA , Choucair A , Flynn PJ , Yung WKA , Prados MD , Bruner JM , Chang SM , Kyritsis AP , Gleason MJ , Hess KR . Phase III randomized study of postradiotherapy chemotherapy with alpha-difluoromethylornithine-procarbazine, N-(2-chloroethyl)-N’-cyclohexyl-N-nitrosurea, vincristine (DFMO-PCV) versus PCV for glioblastoma multiforme. Clin Cancer Res. 2000; 6:3878–84. 11051233

[46] Levin VA , Hess KR , Choucair A , Flynn PJ , Jaeckle KA , Kyritsis AP , Yung WK , Prados MD , Bruner JM , Ictech S , Gleason MJ , Kim HW . Phase III randomized study of postradiotherapy chemotherapy with combination alpha-difluoromethylornithine-PCV versus PCV for anaplastic gliomas. Clin Cancer Res. 2003; 9:981–990. 12631596

[47] Levin VA , Ictech SE , Hess KR . Clinical importance of eflornithine (alpha-difluoromethylornithine) for the treatment of malignant gliomas. CNS Oncol. 2018; 7:CNS16. 10.2217/cns-2017-0031. 29378419 PMC5977277

[48] Koomoa DL , Geerts D , Lange I , Koster J , Pegg AE , Feith DJ , Bachmann AS . DFMO/eflornithine inhibits migration and invasion downstream of MYCN and involves p27Kip1 activity in neuroblastoma. Int J Oncol. 2013; 42:1219–1228. 10.3892/ijo.2013.1835. 23440295 PMC3622674

[49] Koomoa DL , Yco LP , Borsics T , Wallick CJ , Bachmann AS . Ornithine decarboxylase inhibition by alpha-difluoromethylornithine activates opposing signaling pathways via phosphorylation of both Akt/protein kinase B and p27Kip1 in neuroblastoma. Cancer Res. 2008; 68:9825–9831. 10.1158/0008-5472.CAN-08-1865. 19047162 PMC2596629

